# Biomechanical Optimization of the Human Bite Using Numerical Analysis Based on the Finite Element Method

**DOI:** 10.3390/biomimetics10020080

**Published:** 2025-01-28

**Authors:** Maribel González-Martín, Paula Hermida-Cabrera, Aida Gutiérrez-Corrales, Eusebio Torres-Carranza, Gonzalo Ruiz-de-León, Berta García-Mira, Álvaro-José Martínez-González, Daniel Torres-Lagares, María-Ángeles Serrera-Figallo, José-Luis Gutiérrez-Pérez, María Baus-Domínguez

**Affiliations:** 1 Departamento de Estomatología, Facultad de Odontología, Universidad de Sevilla, C/Avicena S/N, 41009 Sevilla, Spain; migm@us.es (M.G.-M.); paulahermidacabrera@gmail.com (P.H.-C.); agcorrales@us.es (A.G.-C.); ruizdeleong@gmail.com (G.R.-d.-L.); danieltl@us.es (D.T.-L.); maserrera@us.es (M.-Á.S.-F.); jlgp@us.es (J.-L.G.-P.); 2 Department of Oral and Maxillofacial Surgery, Virgen del Rocio University Hospital, 41013 Seville, Spain; drtorres@clinicatorrescarranza.es; 3 Departamento de Estomatología, Facultad de Odontología y Medicina, Universidad de Valencia, Avda. Blasco Ibañez 15, 46010 Valencia, Spain; berta.garcia@uv.es; 4 ICEMM S.L.U., Engineering Specialized in FEM and CFD Simulation, C/las Fábricas, Alcorcón, 28923 Madrid, Spain; alvaro.martinez@icemm.es

**Keywords:** bite force, finite element method, dental biomechanics, numerical simulation, dental prosthesis, occlusal force distribution

## Abstract

Biomechanical bite analysis is essential for understanding occlusal forces and their distribution, especially in the design and validation of dental prostheses. Although the finite element method (FEM) has been widely used to evaluate these forces, the existing models often lack accuracy due to simplified geometries and limited material properties. Methods: A detailed finite element model was developed using Abaqus Standard 2023 software (Dassault Systemes, Vélizy-Villacoublay, France), incorporating scanned 3D geometries of mandibular and maxillary bones. The model included cortical and cancellous bones (Young’s modulus: 5.5 GPa and 13.7 GPa, respectively) and was adjusted to simulate bite forces of 220.7 N based on experimental data. Occlusal forces were evaluated using flexible connectors that replicate molar-to-molar interactions, and the stress state was analyzed in the maxillary and mandibular bones. Results: The FEM model consisted of 1.68 million elements, with mesh sizes of 1–1.5 mm in critical areas. Bite forces on the molars were consistent with clinical trials: first molar (59.3 N), second molar (34.4 N), and third molar (16.7 N). The results showed that the maximum principal stresses in the maxillary bones did not exceed ±5 MPa, validating the robustness of the model for biomechanical predictions. Conclusion: The developed model provides an accurate and validated framework for analyzing the distribution of occlusal forces in intact dentures. This approach allows the evaluation of complex prosthetic configurations and their biomechanical impact, optimizing future designs to reduce clinical complications and improve long-term outcomes. The integration of high-resolution FEM models with clinical data establishes a solid foundation for the development of predictive tools in restorative dentistry.

## 1. Introduction

Using implants and prostheses to restore tooth loss is common in contemporary dentistry. Various types have been developed, evaluated, and launched on the market to offer prosthetic, anatomical, aesthetic, and functional solutions to patients who have experienced partial or total tooth loss. Numerous implant systems are now available, backed by the reliability evidenced in various clinical studies [1].

Generally, the dentist’s choice of the type of implant to be installed is derived from the patient’s bone quality and morphology, but the impact it may have on the patient’s bite as a consequence of the redistribution of occlusal loads in the bite is overlooked [1,2].

Understanding occlusion is essential, as it will decrease complications and, thus, patient dissatisfaction, as inadequate contacts cause instability at the dynamic and static occlusion [2].

It is well known that dental implants and teeth do not behave the same way from a physiological point of view. For this reason, the occlusion concepts inherent to natural dentition cannot be applied to implant-supported restorations [3]. The absence of periodontal ligament gives implants lower tactile sensitivity, lower proprioceptive capacity, different load absorption and load distribution behavior, and a lower displacement range than natural teeth (25–100 μm and 56–108 μm, respectively) [4,5].

In these terms, numerous authors have sought to determine the ideal occlusion to protect implant-supported restorations. In 1994, Misch introduced the term “Implanted-protected Occlusion” (IPO), an occlusal scheme that reduces forces at the bone–implant interface [6], which is one of the essential factors for the long-term success of implants [6,7]. In recent years, engineers have joined forces in the search for reliable and reproducible results that guarantee the success of implant-supported restorations. This led to applying the finite element method in dental occlusion in the 1980s [8].

The finite element method (FEM) helps to visualize the location, direction, and magnitude of an applied force, as well as the stress generated in a three-dimensional structure, which cannot be evaluated clinically [2]. It is a method that allows modeling complex structures and analyzing their mechanical properties by digitizing their anatomy, defining material properties (density, elasticity, strength, etc.), and assigning conditions [9].

In the field of implant prosthetics, FEM has been used in recent years to understand and analyze the behavior of different attachments, abutments, and prosthetic configurations [10,11] to understand the biomechanics of each case (single-unit or bridge-on-implant restorations and complete restorations) and, thus, facilitate the clinician’s decision-making [11].

This study aimed to create a simulation model based on the finite element method of the intact mandible and adjusted to real trials, which can be used in the future to predict the distribution of occlusal forces in different prostheses. As a method of evaluation, the occlusal forces obtained in the first, second, and third molars were compared, as these exert more than 95% of the occlusal force.

The T-Scan^®^ provides real-time occlusal balancing data; at the same time, it accurately indicates the relative distribution of bite forces along the dental arch to the total force exerted by the jaws [12]. T-scan system III Version 7.0 has been used by Tarkistani et al. [1] on 132 candidates in order to analyze bite force distribution in subjects with different occlusal characteristics. The distribution of bite pressures of the aforementioned study is used in the current article.

## 2. Materials and Methods

To develop the study, it is necessary to disclose the materials, tools, or resources used and clearly argue the generic theories used.

Below are descriptions of the model used, materials, bone type, and bite forces, as well as the theoretical basis of the study.

### 2.1. Materials

The initial FEM model [13] has hundreds of materials to accurately model the behavior of bone, tendons, muscles, and other soft tissues, both in the elastic and plastic regimes, and failure due to breakage. However, this model does not present sufficient detail for evaluating the behavior of the maxillary bones, which is why these bones are replaced in the original model by others derived from real geometry obtained using 3D scanning.

The definition of the materials used for the mandible and maxillary bones was based on Misch’s classification [14,15], where a Type II bone with a cortical thickness varying between 1.5 mm and 2.0 mm was chosen as the configuration (Table 1).

### 2.2. Occlusion Forces

The occlusal forces resulting from the bite are produced by the contraction of the maxillary muscles that cause the jaw to close. The resulting force results from the contact force between the upper and lower molars if no solid element exists between them.

The evaluation of the tension exerted by the maxillary muscles and tendons is a priori impossible, so we worked in reverse, applying the contact reaction to the teeth and evaluating the force exerted on the elements that simulate the behavior of the maxillary muscles and tendons.

The reaction forces were taken from the studies of Turkistani et al. [1] and Nakano [16].

**Figure 1:**
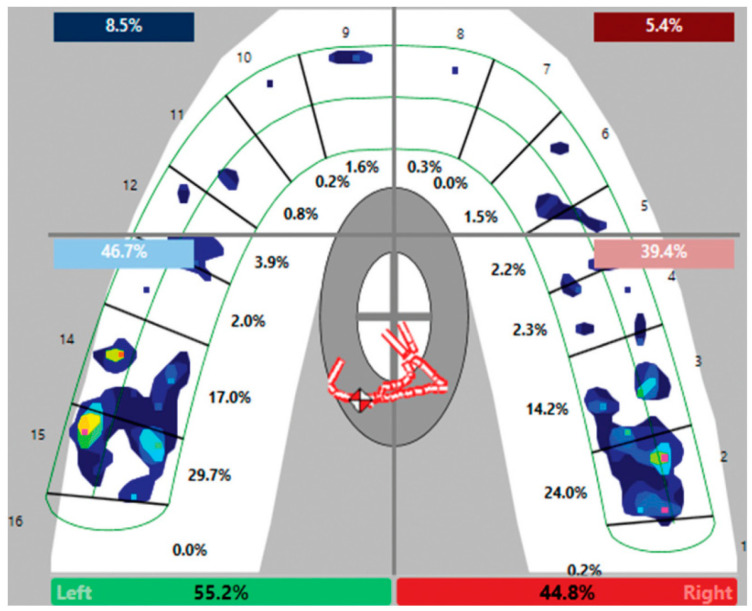
Distribution of bite pressures from the article by Turkistani et al [1]. The colour scale (not included in the reference) is defined from blue to red, where red indicates the highest pressure.

Figure 1 shows the distribution of pressures and the percentage of force according to Turkistani et al. [1].

In accordance with the manufacturer’s instructions (T-Scan^®^ III Version 7.0), participants were seated upright, and the appropriate sensor size was chosen. Ensuring the sensor was kept as parallel as possible to the occlusal plane, participants were asked to complete three chewing cycles before recording the bite force, with the sensitivity adjusted to ensure no more than three “pink peaks” at maximum intercuspation. The average of three consecutive recordings of the maximum bite forces was calculated [1].

For a total occlusal force of 220.7 N [16], the homogenized force values per molar are given in Table 2.

The pressure values shown in Figure 1 for the incisors and canines were distributed to the first molars as they are considered marginal to the forces exerted by the molars.

### 2.3. Finite Element Model

All analyses were conducted using the finite element method using the commercial software Abaqus Standard 2023.

**Figure 2:**
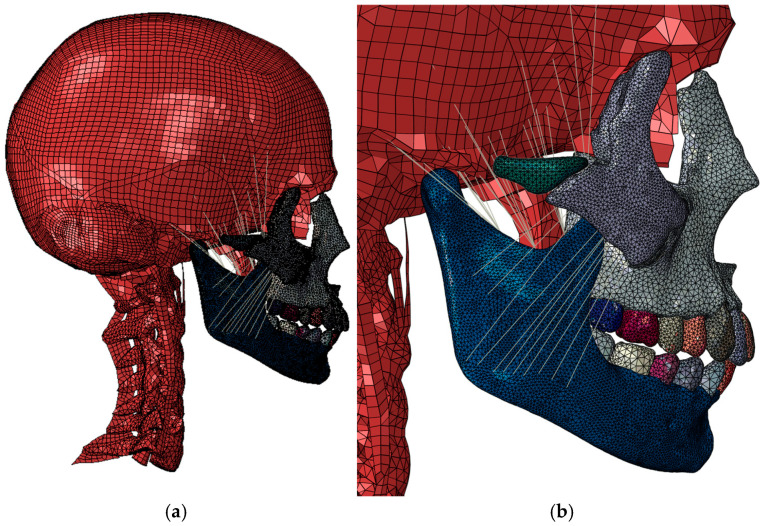

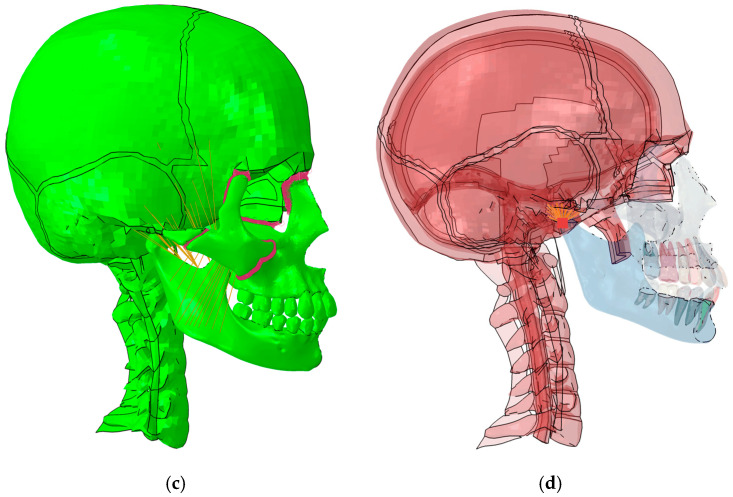
FEM model of bite analysis with intact dentition. (**a**) General lateral view; (**b**) detail of lateral view; (**c**) Tie-type kinematic constraints; (**d**) temporomandibular joint. Different colours have been defined for clarity, and only represent different parts of the model skull. The only exception is image c, where the purple colour represents the articulation between the bones using Tie-type kinematic constraints. In addition, image d has been updated to better show the point of the temporomandibular joint.

The simulation model covers the head and neck up to the C7 cervical vertebra, and all soft tissues except skin, eyes, and ears were retained (Figure 2).

Muscles and tendons were modeled using CONN3D2 connector-type elements, where the connection between the connector and the bone is made using stiffness-free interpolation elements to distribute the load of the connector over an area of influence. The element type used to model the maxilla, zygomatic, mandible, and teeth was C3D10 second-order tetrahedra, with an average mesh size of 1–1.5 mm [17]. The remaining parts were derived from the original model according to [12,17], with first-order C3D8 hexahedral elements for the skull and first-order C3D4 tetrahedra for vertebrae and parts of complex geometry. The total number of elements was 1.68 million elements.

As boundary conditions, all degrees of freedom were restricted to the nodes forming the base of the C7 vertebra.

The connection between the maxilla, zygomatic bone, and the skull was made using Tie-type kinematic restraints, which rigidly connect two surfaces (Figure 2c).

The connection between the mandible and the skull was made using a JOIN connector with free degrees of freedom to rotate, allowing the simulation of the temporomandibular joint (Figure 2d).

### 2.4. Constitutive Model

Given the loading level to which they will be subjected, all materials have been modeled as linear isotropic.

### 2.5. Interaction Between Teeth

The interaction between teeth is produced by contact between the different parts that make up the denture. The parts in an optimal configuration are aligned so that the contact between the parts is optimal. In simulation models, representing this effect is very complex and requires very high mesh densities and the definition of contact stiffnesses between the different parts to obtain the contact forces accurately. This effect is also very local, referring only to the surface of the dental piece, and by applying Saint Venant’s criterion, we can replace the interaction by contact with an interaction based on flexible connectors that transmit the total contact force from one piece to another.

**Figure 3:**
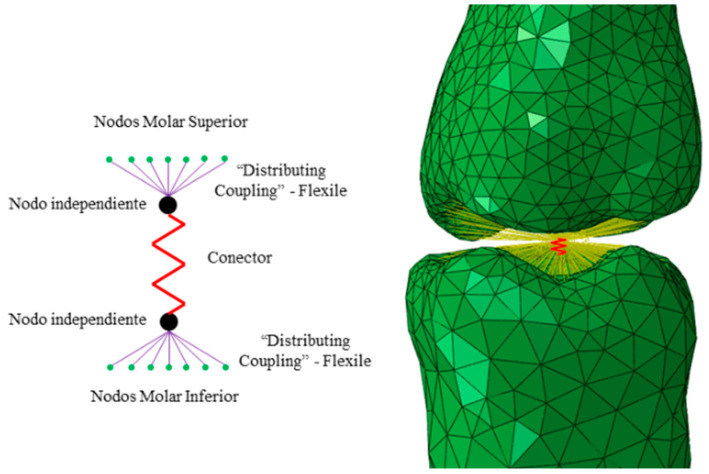
Model of the interaction between teeth. Green is for solid FEM elements. Red for the spring element that model the interaction and yellow for the distributing coupling that distributes the interaction force to the tooth surface.

Figure 3 shows the modeling used to represent the contact between parts.

The contact stiffness between the 3 molars was obtained using an optimization process that minimizes the following objective function:

$$minimize\left(f=\prod_{i=1}^{3}\left({CF}_{FEM,i}-{CF}_{Test,i}\right)\right)$$

where the indices *i* = 1, 2, and 3 refer to the interaction between molars 47–37, 46–36, and 45–35; *CF_FEM,i_* is the contact force obtained from the *FEM* simulation; and *CF_Test,i_* is the contact force defined in Table 2 [1].

## 3. Results

### 3.1. Preload Values in Maxillary Muscles and Tendons

**Figure 4:**
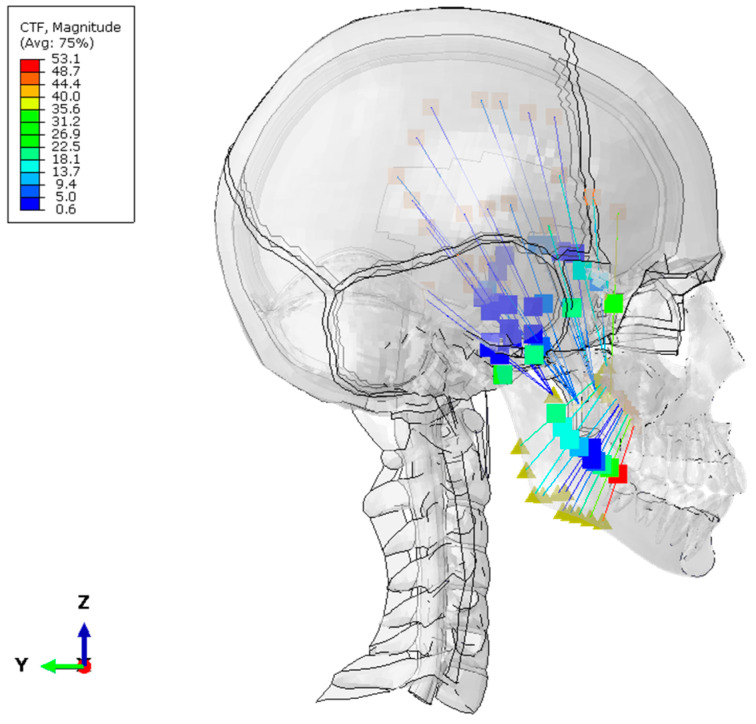
Forces exerted by maxillary muscles and tendons.

The preload values in maxillary muscles and tendons were obtained after applying the process described in the Materials and Methods section (Figure 4).

### 3.2. Occlusion Force Values

**Figure 5:**
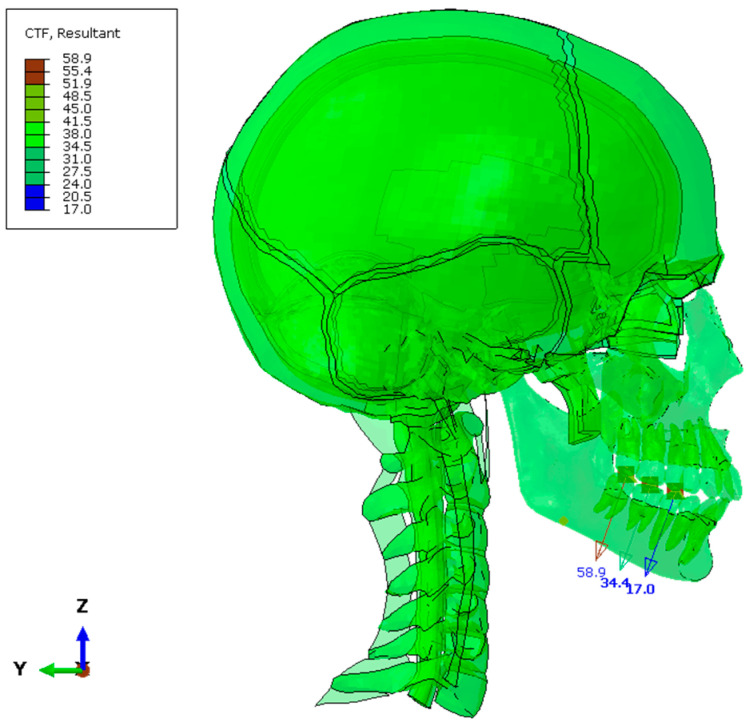
Occlusion forces in 220.7 N bite.

The occlusal forces in the bite are shown in Figure 5. Similarly, the comparative occlusal forces between those obtained from the FEM model and the test values [1] are shown in Table 3.

**Figure 6:**
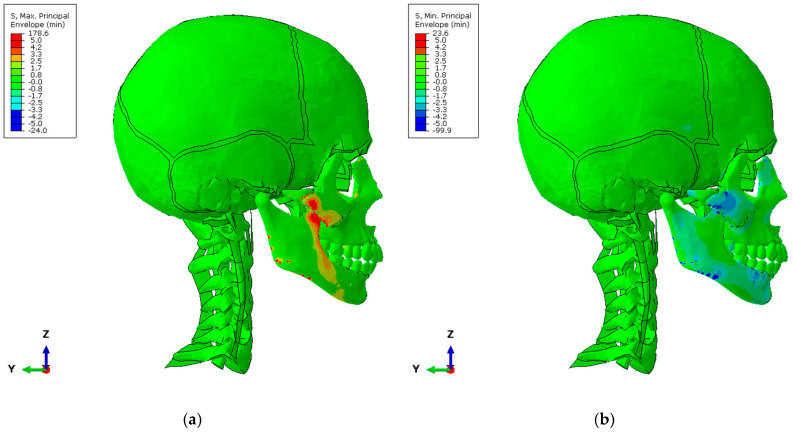
Stress state: (**a**) maximum principal stresses [N/mm^2^]; (**b**) minimum principal stresses [N/mm^2^].

The maximum and minimum principal stress fields are shown in the following figures (Figure 6a,b), where the stress values were filtered to ±5 MPa to smooth out the stress peaks caused by the junction of the connectors modeling muscles and tendons with the jaw bones.

## 4. Discussion

In the present study, the finite element model covered the head and neck up to the C7 vertebra to show the dispersion of forces in the most realistic way. For the same purpose, it was complemented by 3D scans of real bone geometry, thus reflecting high accuracy, all coupled with a total of 1.68 million elements. Depending on their type of union, each structure was linked to adjacent structures using different connectors, depending on the degree of freedom of movement between the anatomical regions or, on the contrary, on their staticity.

The results obtained regarding the distribution of forces at maximum intercuspation in the intact mandible (220.7 N) support the values given in the clinical trial of Turkistani et al. [1] as well as that of Nakano et al. [16]. The greatest masticatory load was on the lower first molar, lower second molar, and lower second premolar, as the present study supports. On the other hand, the distribution of forces was not homogeneous throughout the tooth; physiological and pathological loads (parafunction) mainly affect the amelocemental junction, which is reflected in publications by Dejak et al. [18] and Sender et al. [19]. Both studies yielded these results thanks to the use of the finite element method, which shows the level of precision and power that the FEM has had in the simulation of biomechanical phenomena for more than 30 years, and takes into account current improvements and developments [18].

By understanding how occlusion affects the amelocemental junction, it could be deduced that the prosthesis/implant interface reacts similarly to the distribution of load vectors. To this end, it is essential to highlight the idea reflected by Vaidyanathan [2] about static loads (a point load, e.g., in the case of bruxing patients) and dynamic loads (chewing) and their relation to time. A static load is independent of time, whereas a dynamic load is time-dependent; thus, in the case of the analysis of mastication, understood as a dynamic phenomenon dilated over time, it is necessary to carry out studies that reflect this, since “fatigue stress” is essential to understand not only the behavior of dentures or implant-supported prostheses, but also natural teeth [2].

Linked to dynamic loading, an article by Yeo-Kyeong Lee et al. [20] published in 2020 analyzed the distribution of chewing forces in individuals with different occlusion classes (Angle Class I, complete Class II, and edge-to-edge) using finite elements. It was observed that the distribution of forces was concentrated in the molars, both upper and lower, showing more difference in stress between the upper first molar and lower first molar in individuals with Class II (#16: 4.9 MPa; #46: 2.8 MPa, versus #16: 2.4 MPa; #46: 2.1 MPa), the unit MPa being the value of the Von Mises stress, i.e., the distortion energy [21]. In this case, the force increased when the feed was of a higher consistency. In maximum intercuspation without food, the three cranial models showed comparable and similar force dissipation results. However, in Class I, the central fossa plays a relevant role in stress distribution concerning the dynamic chewing models, in contrast to what is reflected in the present study.

Dental implants do not behave the same way as natural teeth in terms of stress and load response due to the absence of periodontal ligament and the arrangement of their collagen bundles [4,5,21].

A highly illustrative study published by Karimi Dastgerdi et al. [22] reflects the behavior of force distribution in the alveolar bone in the tooth–periodontal ligament–jaw and dental implant–jaw models. It is observed that the stress in terms of the Von Mises stress value is higher in the entire jaw structure in the case of implant carriers than in natural teeth, with the critical points of force distribution in both situations being found in the cervical and apical area (root or implant) [22].

The study by Anitua et al. that was published in 2021 [23] shows that, in terms of load distribution, the essential aspects in implant prostheses are the type of bone in the region analyzed, the diameter of the implant, and the occlusion pattern. In this study, single-unit implant-supported restorations were analyzed, with all implants being 6.5 mm long, placed subcrustal with a 2 mm intermediate abutment in different locations, subjected to pressures of 200 N axially and with an angulation of 30°. The maximum stress was located in the 3.3 mm diameter implants placed in type IV bone (235 MPa) and the lowest level of stress was located in type I bone in implants with a diameter of 4.75 mm (41 MPa). In the long term, this undesired stress at the bone level causes implant failure. The diameter of the implant seems essential, more than the type of bone, since, in the same study, in the case of type IV bone, more trabeculated with a greater capacity to dissipate forces, a larger diameter implant reduced the Von Mises stress value to 68 MPa, so the diameter is a determining factor. This concept that Anitua has been supporting with literature for years [24,25] is also supported by other authors in recent systematic reviews carried out using the finite element method, such as that of Piaopiao Qiu et al. [26], who reached the conclusion after including 40 studies in their review that the diameter has a direct influence on the load distribution in the shoulder area of the implant (something similar to the conclusions obtained previously in studies on teeth [18,19]), and that the length of the implant becomes more important as the bone density decreases.

Moving on to implant-supported restorations, authors reflect in their studies on the behavior of the prosthesis on multiple implants or complete restorations, where the occlusal pattern takes on special relevance. The study by Nurullah Türker et al. [27] is quite representative. In this study, the distribution of forces in implants and soft tissues is analyzed using the finite element method in restorations on all-on-four implants during mastication in different occlusal patterns: bilaterally balanced, mutually protected, group function, lingualized occlusion, and monoplane. All of them were analyzed using maximum intercuspation, lateralization, and protrusive occlusion. In the maxilla, the highest stress level was located in the cortical bone in the occlusal scheme of group function during lateralization movements (15.56 MPa); for the mandible, this maximum value was reflected in the cortical bone in maximum intercuspation of lingualized occlusion (72.75 MPa). Minimum loads were observed for all anatomical structures in the mutually protected occlusion. On the other hand, in a previous study by the same research group, the group function was shown to reduce stress on screws and attachments of multiple implant prostheses [28].

Regarding implant-supported restorations, thanks to the use of finite element analysis, it has been possible to create and reproduce models that allow us to understand that high loads and poor load distribution due to an incorrect occlusal pattern, cantilever location, parafunction, etc., can cause mechanical and biological failures of implants in the long term, as reflected by the European Association for Osseointegration [29,30].

In natural dentition (although it behaves differently), it translates into wear over time of the dentition and allows us to understand the cervical lesions presented in bruxist patients. This is why finite element analysis has applicability in various fields of dentistry, such as endodontics, helping in this case to understand the distribution of stress in a tooth with root canal treatment, temperature changes in them, and resistance to fracture [31].

Finite element analysis has been widely used in dentistry in the last two decades in plenty of different fields.

The current study not only provides data about how forces are distributed in permanent dentition, but also how it affects muscles and tendons, covering structures such as the spinal column up to C7 and the bones composing the skull. When talking about stress distribution, recent papers have used finite element models including restricted areas or just including a recreation of the protheses itself. For example, in the study by Kupprano et al., the model included mimicking the mandibular superstructure consisting of four multi-unit abutments, four bridge screws, and the implant-supported bar connector (ISBC) [32], not taking into account the effect it may have on the rest of the biological structures. Another study by Puengpaiboon [33] included the maxillary left central incisor restored with a single implant-supported crown and the two adjacent teeth.

The results obtained in this intact mandible study are considered highly accurate due to the number of structures and elements included and the support of results demonstrated by previous and subsequent studies on load distribution zones, which are essential for setting up tooth- and implant-supported prostheses.

However, the current study neither provides an answer regarding the behavior of single-borne, bridge-based implant-supported prostheses or complete restorations in patients with an intact mandible, nor how the distribution of forces in the occlusion given to them affects the anatomical structures included in the study. This is relevant for future research, since, thanks to the finite element method, various situations can be recreated, as has already been demonstrated in the literature.

## 5. Conclusions

This study has demonstrated the ability of the finite element method-based model to accurately simulate bite behavior in an intact dentition under a total occlusion force of 220.7 N. The results obtained, with forces distributed mainly in the first molar (59.3 N), second molar (34.4 N), and third molar (16.7 N), are highly consistent with previous clinical data, validating the accuracy and applicability of the model. Furthermore, the maximum stresses observed in the maxillary bones remained within physiologically reasonable ranges, highlighting the model’s reliability for evaluating complex prosthetic configurations.

The presented model not only facilitates the prediction of occlusal force distributions under different clinical scenarios, but also provides a solid framework for optimizing the design of dental prostheses, improving both the functionality and durability of rehabilitations. This approach contributes significantly to clinical decision-making by allowing the biomechanical impacts of different implant and prosthetic configurations to be evaluated before implementation.

## Figures and Tables

**Table 1:** Initial data used in the numerical models for bone type II [4].

	Young’s Modulus (GPa)	Poisson’s Ratio	Density (g/cm^3^)
Cancellous bone	5.5	0.3	2.12
Cortical bone	13.7	0.3	2.12

**Table 2:** Occlusion forces per molar for a total load of 220.7 N.

Molars	% Load	Force [N] Per Mole
47–37	53.7%	59.3
46–36	31.2%	34.4
45–35	15.1%	16.7

**Table 3:** Comparative occlusal forces per molar for a total load of 220.7 N.

**Molars**	**Test**	**FEM**
47–37	59.3 N	58.9 N
46–36	34.4 N	34.4 N
45–35	16.7 N	17.0 N

## Data Availability

This manuscript contains all necessary data regarding the research. If any reader has any questions, we recommend that they contact the corresponding authors.
